# Allogeneic cord blood regulatory T cells decrease dsDNA antibody and improve albuminuria in systemic lupus erythematosus

**DOI:** 10.3389/fimmu.2023.1217121

**Published:** 2023-09-05

**Authors:** Mi-Ae Lyu, Ximing Tang, Joseph D. Khoury, Maria Gabriela Raso, Meixian Huang, Ke Zeng, Mitsutaka Nishimoto, Hongbing Ma, Tara Sadeghi, Christopher R. Flowers, Simrit Parmar

**Affiliations:** ^1^ Department of Lymphoma/Myeloma, The University of Texas M.D. Anderson Cancer Center, Houston, TX, United States; ^2^ Department of Translational Molecular Pathology, The University of Texas M.D. Anderson Cancer Center, Houston, TX, United States; ^3^ Department of Hematopathology, The University of Texas M.D. Anderson Cancer Center, Houston, TX, United States; ^4^ Cellenkos Inc., Houston, TX, United States

**Keywords:** umbilical cord blood (UCB), allogeneic, regulatory T cells (Tregs), systemic lupus erythematosus (SLE), lupus nephritis (LN), dsDNA antibodies, albuminuria

## Abstract

**Background:**

Lupus nephritis (LN) constitutes the most severe organ manifestations of systemic lupus erythematosus (SLE), where pathogenic T cells have been identified to play an essential role in ‘helping’ B cells to make autoantibodies and produce inflammatory cytokines that drive kidney injury in SLE. Regulatory T cells (Tregs), responsible for decreasing inflammation, are defective and decreased in SLE and have been associated with disease progression. We hypothesize that treatment with allogeneic, healthy Tregs derived from umbilical cord blood (UCB) may arrest such an inflammatory process and protect against kidney damage.

**Methods:**

UCB-Tregs function was examined by their ability to suppress CellTrace Violet-labeled SLE peripheral blood mononuclear cells (PBMCs) or healthy donor (HD) conventional T cells (Tcons); and by inhibiting secretion of inflammatory cytokines by SLE PBMCs. Humanized SLE model was established where female Rag2^-/-^γc^-/-^ mice were transplanted with 3 × 10^6^ human SLE-PBMCs by intravenous injection on day 0, followed by single or multiple injection of UCB-Tregs to understand their impact on disease development. Mice PB was assessed weekly by flow cytometry. Phenotypic analysis of isolated cells from mouse PB, lung, spleen, liver and kidney was performed by flow cytometry. Kidney damage was assessed by quantifying urinary albumin and creatinine secretion. Systemic disease was evaluated by anti-dsDNA IgG Ab analysis as well as immunohistochemistry analysis of organs. Systemic inflammation was determined by measuring cytokine levels.

**Results:**

*In vitro*, UCB-Tregs are able to suppress HD Tcons and pathogenic SLE-PBMCs to a similar extent. UCB-Tregs decrease secretion of several inflammatory cytokines including IFN-γ, IP-10, TNF-α, IL-6, IL-17A, and sCD40L by SLE PBMCs in a time-dependent manner, with a corresponding increase in secretion of suppressor cytokine, IL-10. *In vivo*, single or multiple doses of UCB-Tregs led to a decrease in CD8^+^ T effector cells in different organs and a decrease in circulating inflammatory cytokines. Improvement in skin inflammation and loss of hair; and resolution of CD3^+^, CD8^+^, CD20^+^ and Ki67^+^ SLE-PBMC infiltration was observed in UCB-Treg recipients with a corresponding decrease in plasma anti-double stranded DNA IgG antibody levels and improved albuminuria.

**Conclusions:**

UCB-Tregs can decrease inflammatory burden in SLE, reduce auto-antibody production and resolve end organ damage especially, improve kidney function. Adoptive therapy with UCB-Tregs should be explored for treatment of lupus nephritis in the clinical setting.

## Highlights

- UCB-Tregs suppress pathogenic SLE cells, decrease CD19^+^B cells and monocytes, increase IL-10, and decrease IFN-γ, IP-10, TNF-α, IL-6, IL-17A, and sCD40L.
- UCB-Tregs decrease CD8^+^T and CD20^+^B cell tissue infiltration, inflammatory cytokines, anti-dsDNA IgG Ab, and albuminuria in lupus xenografts.

## Introduction

Systemic lupus erythematosus (SLE) is known to be a B-cell-mediated autoimmune disorder with multi-organ involvement, including skin rash, pulmonary fibrosis, joint pain, neurological dysfunction, vasculitis, and renal failure (1, 2). Recent data support the contribution of autoreactive, pathogenic T cells in the perpetuation of the autoimmune process including autoantibody production and tissue inflammation in SLE (3–5). In fact, adoptive transfer of T cells overexpressing LFA-1 can induce lupus-like disease including glomerulonephritis in naïve recipient mice (6).

Lupus nephritis (LN), a fatal complication of SLE, is thought to be caused by an inflammatory response to immunogenic, endogenous chromatin and driven by the autoreactive leukocytes, immune complexes (ICs), and IL-17-producing T helper 17 cells (TH17 cells) in collaboration with various cytokines, chemokines, and growth factors, where the treatment usually consists of immunosuppressive therapy including steroids (7).

CD4^+^CD25^+^CD127^low^ regulatory T cells (Tregs) expressing the lineage-specific transcription factor forkhead box P3 (FoxP3) regulate the activation and expansion of auto-reactive T cells and other harmful immune cells in the peripheral lymphatic organs and prevent and control inflammation and autoimmunity (8). The FoxP3 protein is the master regulator for development and function of CD4^+^CD25^+^CD127^low^ Treg cells, where mutations of FoxP3 gene impair their development and function that may result in severe autoimmune disease (9–11). Previously, it has been reported that SLE patients have a lower percentage of CD4^+^CD25^+^CD127^low^ Tregs when compared to a healthy population, and CD4^+^CD25^+^CD127^low^ Tregs derived from SLE patients show defect in their suppressor function (12–15) and are functionally exhausted (16, 17). Additionally, the SLE pathogenic T effector (Teff) cells develop resistance to CD4^+^CD25^+^CD127^low^ Treg-induced suppression (18). Such a “double whammy” leads to a heavily inflammatory microenvironment and a continuous loop of tissue destruction resulting in end organ damage, especially kidney damage (19).

Findings from these studies suggest that adoptive therapy with CD4^+^CD25^+^CD127^low^ Tregs may represent a potential therapeutic strategy for treating inflammatory processes in SLE. We have previously shown that adoptive therapy with allogeneic, umbilical cord blood (UCB)-derived CD4^+^CD25^+^CD127^low^ Tregs can prevent graft vs. host disease (20, 21), resolve lung inflammation (22), treat COVID-19-associated acute respiratory distress syndrome and multi-organ failure (23), and show early survival benefit (24).

We now hypothesize that CD4^+^CD25^+^CD127^low^ UCB-Tregs can treat LN, leveraging their unique properties including (i) lack of plasticity when exposed to inflammatory micro-environments; (ii) no requirement for HLA matching with the recipients; (iii) long shelf life of the cryopreserved cells; and (iv) immediate product availability for on-demand treatment (22).

## Materials and methods

### SLE and healthy donor PBMCs

Human SLE-peripheral blood mononuclear cells (SLE-PBMCs) (ASTARTE Biologics, Bothell, WA, USA) or healthy donor PBMCs (HD-PBMCs) (Gulf Coast Blood Bank, Houston, TX, USA) were purified using Lymphoprep (StemCell Technologies, Vancouver, BC, Canada) and then cultured in X-VIVO 15 medium (Lonza Biowhittaker, Morristown, NJ, USA) with 10% fetal bovine serum (FBS) (Thermo Fisher Scientific, Waltham, USA), 2 mM L-glutamine (Sigma-Aldrich, St Louis, MO, USA), 1% penicillin–streptomycin (Thermo Fisher Scientific), and 1,000 IU/ml IL-2 (Clinigen Inc., Yardley, PA, USA) in the presence of CD3/CD28 beads (Thermo Fisher Scientific) for 3–7 days.

### UCB-Treg cell isolation and *ex vivo* expansion

CD4^+^CD25^+^CD127^low^ Treg cells were isolated from UCB units and cultured and cryopreserved as described previously (22). Additionally, frozen CD4^+^CD25^+^CD127^low^ UCB-Tregs were provided by Cellenkos Inc (Houston, TX, USA). Cryopreserved CD4^+^CD25^+^CD127^low^ UCB-Tregs were thawed and cultured in X-VIVO 15 medium supplemented with 10% FBS, 2 mM L-glutamine, 1% penicillin–streptomycin, and 1,000 IU/ml IL-2 for 3–7 days.

### Flow cytometry analysis

APC-eFluor 780-conjugated mouse anti-human CD45 antibody (Ab) (HI30), Alexa Fluor-532-conjugated mouse anti-human CD3 Ab (UCHT1), FITC-conjugated mouse anti-human CD3 Ab (UCHT1), PerCP-Cyanine5.5-conjugated mouse anti-human CD8a Ab (RTA-T8), Super Bright 600-conjugated mouse anti-human CD19 Ab (SJ25C1), PE-conjugated mouse anti-human CD25 Ab (BC96), PE-Cy5-conjugated mouse anti-human CD127 Ab (eBioRDR5), APC-conjugated mouse anti-human CD56 Ab (CMSSB), FITC-conjugated mouse anti-human CD16 Ab (eBioCB16(CB16)), PerCP-eFluor 710-conjugated mouse anti-human CD14 Ab (61D3), PE-Cy7-conjugated mouse anti-human HLA-DR Ab (LN3), and LIVE/DEAD™ fixable Blue dye were purchased from Thermo Fisher Scientific. BV650-conjugated mouse anti-human CD4 Ab (L200), BV510-conjugated mouse anti-human CD8 Ab (RPA-T8), PE-CF594-conjugated mouse anti-human CD27 Ab (M-T271), Alexa Fluor 700-conjugated mouse anti-human IgD Ab (IA6-2), BV421-conjugated mouse anti-human CD62L Ab (SK11), Alexa Fluor 647-conjugated Armenian hamster anti-Helios Ab (22F6), Alexa Fluor 647-conjugated mouse anti-human FoxP3 Ab (259D/C7), and PerCP-Cy5.5-conjugated mouse anti-human FoxP3 Ab (236A/E7) were purchased from BD Biosciences. Pacific Blue-conjugated mouse anti-mouse CD45.1 Ab (A20) was purchased from SouthernBiotech (Birmingham, AL, USA). The antibodies per test were used according to the vendor’s instruction.

Events in the t-distributed stochastic neighbor embedding (t-SNE) were overlaid with manually gated lymphocytes, CD3^+^CD19^-^, CD4^+^ T, CD4^+^CD25^+^ T, CD4^+^CD25^+^CD127^low^ Treg, CD4^+^CD8^+^ T, CD8^+^ T, CD3^-^CD19^+^ B, CD27^-^IgD^-^ double negative (DN) B, CD27^+^IgD^-^ Memory B, CD27^-^IgD^+^ Naïve B, CD27^+^IgD^+^ Plasma, CD56^+^ NK cells, and CD14^+^ monocytes and displayed for all treatments in the concatenated file. Stained cells were acquired on Cytek Aurora flow cytometer (Cytek Biosciences, Fremont, CA, USA) or a BD LSRFortessa flow cytometer (BD Biosciences) and analyzed using FlowJo software (FlowJo, LLC, Ashland, OR, USA).

### Suppression assay

CD4^+^CD25^-^ conventional T cells (Tcons) or SLE-PBMCs were stained with CellTrace Violet (CTV) (Thermo Fisher Scientific) following the manufacturer’s instruction. CTV-labeled Tcons or SLE-PBMCs were co-cultured with different ratios of unlabeled CD4^+^CD25^+^CD127^low^ UCB-Tregs in the presence of CD3/CD28 beads. Proliferation of CTV-labeled Tcons or SLE-PBMCs was assessed by LSRFortessa Cell Analyzer as described previously (22).

### Detection of human cytokines in cell culture supernatants

HD-PBMCs, SLE-PBMCs, or CD4^+^CD25^+^CD127^low^ UCB-Tregs, or a combination were cultured for 3 or 7 days; culture supernatants were collected for cytokine analysis; and human IL-10, IFN-γ, IP-10, TNF-α, IL-6, and IL-17A were assessed using Human Cytokine ELISA kits (Thermo Fisher Scientific) according to the manufacturer’s instructions. Levels of sCD40L, IL-1α, and IL-21 were measured using Human Cytokine/Chemokine 71-plex Discovery Assay Array kit (Eve Technologies, Calgary, AB, Canada).

### SLE xenograft model

Animal procedures were performed according to an approved protocol by MD Anderson Cancer Center IACUC. *Rag2/IL2rg* compound mutant mice (Rag2^-/-^ γc^-/-^ mice) were purchased from The Jackson Laboratory (Bar Harbor, ME, USA) at 4 weeks of age and were transplanted with 3 × 10^6^ SLE-PBMCs by intravenous tail vein (t.v.) injection (25). After mice displayed human immune cells, 10 × 10^6^ CD4^+^CD25^+^CD127^low^ UCB-Treg cells were injected intravenously on day 7 for single treatment (*n* = 3) or on days 30, 35, 45, and 51 for multiple treatment (*n* = 7). PB SLE cells as well as production of anti-double-stranded DNA IgG antibody were detected in SLE xenografts. Mice were assessed weekly for the reconstitution level of human immune cells by flow cytometry of the peripheral blood. Mice were monitored twice per week for weight loss and survival. At the time of euthanasia, organs of SLE xenografts were aseptically harvested, homogenized, and filtered using a nylon mesh to obtain single-cell suspensions. After RBC lysis, tissue cells were collected by centrifugation and phenotypic analysis of cells was performed by analysis of surface or intracellular markers. Data acquired by LSRFortessa Cell Analyzer (BD Biosciences) were analyzed with BD FACSDiva 8.0.1 software and FlowJo software (FlowJo LLC, Ashland, Oregon, USA).

### Detection of urinary albumin in SLE xenografts

Urine samples were collected every other week for the assessment of kidney function in SLE xenografts and the levels of albumin and creatinine were assessed using the Exocell Albumin M assay kit (Ethos Biosciences, Logan Township, NJ, USA) according to the manufacturer’s instructions.

### Detection of anti-human double-stranded DNA IgG antibody in SLE xenografts

Plasma samples were collected weekly from the EDTA-treated peripheral blood of untreated and *ex vivo* expanded CD4^+^CD25^+^CD127^low^ UCB-Tregs-infused SLE mice. The level of anti-human double-stranded DNA IgG Ab in the plasma was assessed using the Abnova assay kit (Abnova, Neihu District, Taipei City, Taiwan) according to the manufacturer’s instruction.

### Detection of human cytokine/chemokine in the plasma of SLE xenografts

Plasma samples were collected weekly from the EDTA-treated PB of SLE xenografts and levels of inflammatory cytokines were measured using Human cytokine 42-plex Discovery assay kit (Eve Technologies).

### Histopathology and immunohistochemistry

Harvested organs were fixed with 10% buffered formalin and embedded in paraffin for processing into 5-μm tissue sections. De-paraffinized and rehydrated tissue sections were stained with Hematoxylin & Eosin, and evaluated by an institution pathologist, who was blinded to the treatment arms. For immunohistochemistry (IHC), de-paraffinized and rehydrated tissue sections were subjected to heat-mediated antigen retrieval with sodium citrate buffer (pH 6), permeabilization, and blocking prior to staining with primary antibodies including human CD3 (Cat #A0452, Clone F7.2.38) (DAKO, Santa Clara, CA, USA), CD4 (Cat #CD4-368-L-CE, Clone 4B12) (Leika Biosystems Inc., Buffalo, Grove, IL, USA), CD8 (Cat #MS-457s, Clone C8/144B) (Thermo Fisher Scientific), CD20 (Cat #MO755, Clone L26) (DAKO), and Ki-67 (Cat #M7240, Clone MIB-1) (DAKO). Appropriate horseradish peroxidase-conjugated secondary antibodies were used and the stained tissue slides were analyzed using Aperio ImageScope (Leica Biosystems Inc., Buffalo Grove, IL, USA). IHC images were analyzed at ×40 magnification using HALO 3.3 software (India Labs, Albuquerque, NM, USA), and H-score was defined by the percentage of strongly positive stain × 3 + moderately positive stain × 2 + weakly positive stain × 1. A final value of 0–300 was also calculated using HALO software.

### Statistical analysis

All statistical analyses were done with GraphPad Prism 9 software (San Diego, CA, USA). Data are presented as mean ± SEM. *p*-values were obtained using one or two-way analysis of variance (ANOVA) with Tukey multiple comparison test, *F*-test, or two-tailed unpaired *t-*test with 95% confidence interval for evaluation of statistical significance compared with the untreated controls. *p* < 0.05 was considered statistically significant.

## Results

### CD4^+^CD25^+^CD127^low^ UCB-Tregs suppress SLE-PBMCs proliferation and decrease CD19^+^ B cells

**Figure 1:**
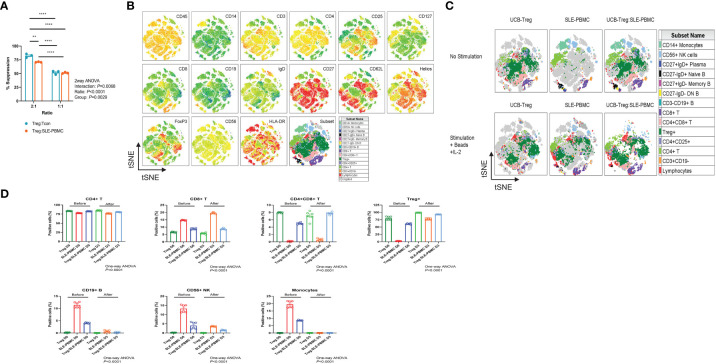
CD4^+^CD25^+^CD127^low^ UCB-Tregs suppress SLE-PBMC and shift their cell population distribution. **(A)** Functional analysis of *ex vivo*-expanded day 14 CD4^+^CD25^+^CD127^low^ UCB-Tregs on suppression of Tcon cells from healthy donor and SLE-PBMCs. Two-way ANOVA demonstrated that ratio (*p* < 0.0001), group (*p* = 0.0029), and interaction (*p* = 0.0068) between UCB-Treg : Tcon and UCB-Treg : SLE-PBMC were statistically significant. Data are presented as mean ± SEM (*n* = 3). *p* < 0.05 was considered statistically significant. **p < 0.01; ****p < 0.0001 by two-way ANOVA with Tukey multiple comparison tests. **(B)** Expression levels of surface and intracellular markers on tSNE map. Unstimulated or stimulated CD4^+^CD25^+^CD127^low^ UCB-Tregs, SLE-PBMCs, or CD4^+^CD25^+^CD127^low^ UCB-Treg plus SLE-PBMC co-cultures (1:1) with CD3/CD28 plus IL-2 were stained with Live/Dead dye, CD45, CD14, CD3, CD4, CD25, CD127, CD8, CD19, IgD, CD27, CD62L, Helios, FoxP3, CD56, and HLA-DR antibodies. Stained live CD45^+^ cells from all six treatments were gated, down-sampled to 10,000 cells per sample which were concatenated. tSNE was run on six samples and the resulting tSNE plots were displayed expression intensities of surface and intracellular markers for all treatments in the concatenated file. **(C)** Subset analysis on tSNE map. Events in the tSNE embeddings were overlaid with manually gated lymphocytes, CD3^+^CD19^-^ T, CD4^+^ T, CD4^+^CD25^+^ T, CD4^+^CD25^+^CD127^low^ Treg, CD4^+^CD8^+^ T, CD8^+^ T, CD3^-^CD19^+^ B, CD27^-^IgD^-^ DN B, CD27^+^IgD^-^ Memory B, CD27^-^IgD^+^ Naïve B, CD27^+^IgD^+^ Plasma, CD56^+^ NK cells, and CD14^+^ monocytes and displayed for all treatments in the concatenated file. Stained cells were acquired on a Cytek Aurora flow cytometer and analyzed using FlowJo software. **(D)** Quantification analysis of subsets. Unstimulated or stimulated CD4^+^CD25^+^CD127^low^ UCB-Tregs, SLE-PBMCs, or CD4^+^CD25^+^CD127^low^ UCB-Treg plus SLE-PBMC co-cultures (1:1) with CD3/CD28 plus IL-2 were stained with Live/Dead dye, CD45, CD14, CD3, CD4, CD25, CD127, CD8, CD19, IgD, CD27, CD62L, Helios, FoxP3, CD56, and HLA-DR antibodies. CD4^+^ T, CD8^+^ T, CD4^+^CD8^+^ T, CD4^+^CD25^+^CD127^low^ Treg, CD3^-^CD19^+^ B, CD56^+^ NK cells, and CD14^+^ monocytes were quantified. Data are presented as mean ± SEM (*n* = 6). *p* < 0.05 was considered statistically significant. *p* < 0.0001 by one-way ANOVA test.

UCB-Tregs phenotype was consistent with CD4^+^CD25^high^CD127^low^ FoxP3^+^ (22). Since pathogenic SLE PBMCs may be resistant to Treg-mediated suppression (18), we compared the ability of CD4^+^CD25^+^CD127^low^ UCB-Tregs to suppress proliferation of target cells. As shown in **Figure 1A**, no differences were observed in the ability of the CD4^+^CD25^+^CD127^low^ UCB-Treg cells to suppress HD-Tcons vs. SLE-PBMCs at a 1:1 ratio. Initial analysis of the expression levels of surface and intracellular markers, including CD45, CD14, CD3, CD4, CD25, CD127, CD8, CD19, IgD, CD27, CD62L, Helios, FoxP3, CD56, and HLA-DR marker on the tSNE map, allowed for grouping of the cell populations into monocytes and lymphocytes including CD4^+^T, CD8^+^T, and CD4^+^CD8^+^T cells; CD4^+^CD25^+^CD127^low^Treg cells; CD19^+^B cells; and CD56^+^NK cells (**Figure 1B**). As shown in **Figures 1C, D**, CD4^+^CD25^+^CD127^low^ UCB-Tregs decrease the inflammatory cell population of SLE-PBMCs. After stimulation with CD3/CD28 beads and IL-2 for 3 days, an increase in the percentage of the CD4^+^CD25^+^CD127^low^-expressing Treg cells was observed in SLE-PBMCs, whereas their distribution rearrangement was observed in CD4^+^CD25^+^CD127^low^ UCB-Tregs where an overall decrease in the CD8^+^ only expressing T cells with an actual increase in the CD4^+^ and CD8^+^ co-expressing cells was observed in UCB-Tregs : SLE-PBMCs co-culture. Additionally, a decrease in CD19^+^ B cells, CD56^+^ NK cells, and CD14^+^ monocytes was also observed.

### CD4^+^CD25^+^CD127^low^ UCB-Tregs increase IL-10 and decrease inflammation

**Figure 2:**
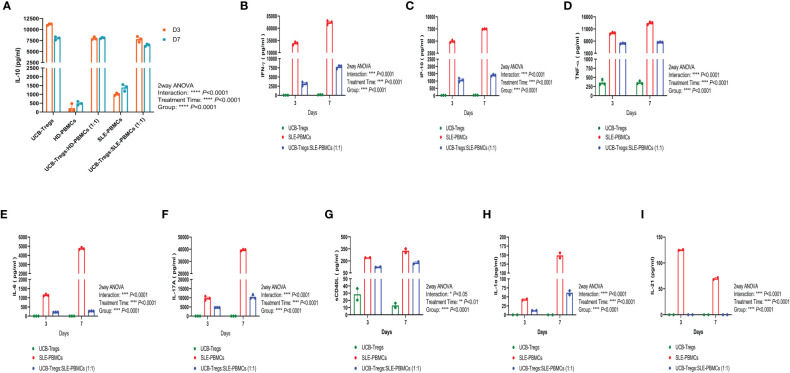
CD4^+^CD25^+^CD127^low^ UCB-Tregs increase IL-10 secretion and decrease inflammatory cytokines in co-culture with SLE-PBMCs. CD4^+^CD25^+^CD127^low^ UCB-Tregs, HD-PBMCs, SLE-PBMCs, or UCB-Tregs plus SLE-PBMC co-cultures (1:1) were seeded at 1 × 10^6^ cells per well in six-well plates and were stimulated with Human T-Activator CD3/CD28 in a 1 cell:1 bead ratio in X-VIVO 15 medium supplemented with 10% FBS, 2 mM L-glutamine, 1% penicillin–streptomycin, and 1,000 IU/ml IL-2. After 3 days or 7 days, cell culture supernatants were collected for cytokine analysis. Production levels of human IL-10 **(A)**, IFN-γ **(B)**, IP-10 **(C)**, TNF-α **(D)**, IL-6 **(E)**, and IL-17A **(F)** in the cell culture supernatants were assessed using Human Cytokine ELISA kits according to manufacturer’s instructions and production levels of sCD40L **(G)**, IL-1α **(H)**, and IL-21 **(I)** were measured using human cytokine/chemokine 71-plex discovery assay array. Data are presented as mean ± SEM (*n* = 2–3). *p* < 0.05 was considered statistically significant. *p < 0.05, **p < 0.01; ***p < 0.001; ****p < 0.0001 by two-way ANOVA with Tukey multiple comparison tests.

CD4^+^CD25^+^CD127^low^ Tregs can suppress Teff cell proliferation by secreting inhibitory cytokine IL-10 (26). As shown **Figure 2A**, high levels of soluble IL-10 were detected in CD4^+^CD25^+^CD127^low^ UCB-Tregs supernatant at days 3 and 7 (11,169 ± 118 pg/ml and 8,019 ± 221 pg/ml, respectively), whereas minimal IL-10 secretion was detected in HD-PBMC- or SLE-PBMC-alone supernatant at day 3 (223 ± 130 pg/ml and 1,010 ± 38 pg/ml, respectively) and day 7 (494 ± 59 pg/ml and 1,391 ± 111 pg/ml, respectively). Co-culture of HD-PBMCs or SLE-PBMCs with CD4^+^CD25^+^CD127^low^ UCB-Tregs increased IL-10 production on day 3 (7,995 ± 182 pg/ml and 7,802 ± 372 pg/ml, respectively) and day 7 (8,086 ± 86 pg/ml and 6,466 ± 152 pg/ml) (two-way ANOVA *p* < 0.0001).

As shown in **Figures 2B–I**, CD4^+^CD25^+^CD127^low^ UCB-Tregs were able to suppress several inflammatory cytokines secreted by SLE-PBMCs in their co-culture supernatants on days 3 and 7, including the following: (i) IFN-γ: Significantly higher IFN-γ levels of 29,264 ± 1,867 pg/ml and 72,080 ± 2,379 pg/ml were secreted by SLE-PBMCs alone at days 3 and 7, respectively, when compared to CD4^+^CD25^+^CD127^low^ UCB-Tregs alone levels of 32 ± 3 pg/ml and 149 ± 16 pg/ml at the same time points (*p* < 0.0001). Significantly decreased IFN-γ levels of 3,155 ± 310 pg/ml at 3 days (*p* = 0.0002) and 7,767 ± 271 pg/ml at 7 days (*p* < 0.0001, **Figure 2B**) were detected in their co-culture supernatants. (ii) IP-10: Significantly higher IP-10 levels of 4,941 ± 140 pg/ml and 7,505 ± 33 pg/ml were secreted by SLE-PBMCs alone at days 3 and 7, respectively, when compared to CD4^+^CD25^+^CD127^low^ UCB-Tregs alone levels of 22 ± 2 pg/ml and 33 ± 2 pg/ml at the same time points (*p* < 0.0001). Addition of CD4^+^CD25^+^CD127^low^ UCB-Tregs to the proliferating SLE-PBMCs significantly decreased the secreted IP-10 levels to 1,068 ± 77 pg/ml at 3 days (*p* < 0.0001) and 1,340 ± 35 pg/ml IP-10 at 7 days (*p* < 0.0001, **Figure 2C**). (iii) TNF-α: Significantly higher TNF-α levels of 9,342 ± 202 pg/ml at 3 days and 13,426 ± 333 pg/ml at 7 days were secreted by SLE-PBMCs when compared to CD4^+^CD25^+^CD127^low^ UCB-Tregs alone levels of 358 ± 45 pg/ml and 355 ± 24 pg/ml, at the corresponding time points (*p* < 0.0001). Addition of CD4^+^CD25^+^CD127^low^ UCB-Tregs to the proliferating SLE-PBMCs significantly decreased the secreted TNF-α levels to 5,071 ± 97 pg/ml at 3 days (*p* < 0.0001) and 5,564 ± 50 pg/ml at 7 days (*p* < 0.0001, **Figure 2D**). (iv) IL-6: Significantly higher IL-6 levels of 1,150 ± 30 pg/ml at 3 days and 4,747 ± 67 pg/ml IL-6 at 7 days were secreted by SLE-PBMCs when compared to CD4^+^CD25^+^CD127^low^ UCB-Tregs alone levels of 0 pg/ml for both the corresponding time points (*p* < 0.0001). The addition of CD4^+^CD25^+^CD127^low^ UCB-Tregs to the proliferating SLE-PBMCs significantly decreased the secreted IL-6 levels to 213 ± 3 pg/ml at 3 days (*p* < 0.0001) and 273 ± 5 pg/ml at 7 days (*p* < 0.0001, **Figure 2E**). (v) IL-17A: Significantly higher IL-17A levels of 9,770 ± 616 pg/ml at 3 days and 39,402 ± 374 pg/ml at 7 days were secreted by SLE-PBMCs when compared to CD4^+^CD25^+^CD127^low^ UCB-Tregs alone levels of 14 ± 1 pg/ml and 13 ± 2 pg/ml at the corresponding time points (*p* < 0.0001). The addition of CD4^+^CD25^+^CD127^low^ UCB-Tregs to the proliferating SLE-PBMCs significantly decreased the secreted IL-17A levels to 4,705 ± 43 pg/ml at 3 days (*p* = 0.0012) and 10,438 ± 668 pg/ml at 7 days (*p* < 0.0001, **Figure 2F**). (vi) sCD40L: Significantly higher sCD40L levels of 241 ± 1 pg/ml at 3 days and 327 ± 24 pg/ml sCD40L at 7 days were secreted by SLE-PBMCs when compared to CD4^+^CD25^+^CD127^low^ UCB-Tregs alone levels of 29 ± 8 pg/ml and 13 ± 3 at the corresponding time points (*p* < 0.0001). The addition of CD4^+^CD25^+^CD127^low^ UCB-Tregs to the proliferating SLE-PBMCs significantly decreased the secreted sCD40L level to 124 ± 3 pg/ml at 3 days (*p* = 0.0006) and 177 ± 13 pg/ml at 7 days (*p* = 0.0336; **Figure 2G**). (vii) IL-1α: CD4^+^CD25^+^CD127^low^ UCB-Tregs did not produce IL-1α. SLE-PBMCs alone produced 43 ± 2 pg/ml at 3 days and 149 ± 7 pg/ml IL-1α at 7 days (*p* = 0.0046, when compared to CD4^+^CD25^+^CD127^low^ UCB-Tregs). The addition of CD4^+^CD25^+^CD127^low^ UCB-Tregs to the proliferating SLE-PBMCs significantly decreased the secreted IL-1α levels to 12 ± 0 pg/ml at 3 days (*p* = 0.0029) and 61 ± 6 pg/ml at 7 days (*p* = 0.0106; **Figure 2H**). (viii) IL-21: CD4^+^CD25^+^CD127^low^ UCB-Tregs did not produce IL-21. SLE-PBMCs alone produced 126 ± 1 pg/ml at 3 days and 70 ± 2 pg/ml IL-21 at 7 days. The addition of CD4^+^CD25^+^CD127^low^ UCB-Tregs to the proliferating SLE-PBMCs led to undetectable IL-21 levels at day 3 (*p* < 0.0001) and day 7 (*p* = 0.0007; **Figure 2I**).

### Single injection of CD4^+^CD25^+^CD127^low^ UCB-Tregs slows SLE development *in vivo*

**Figure 3:**
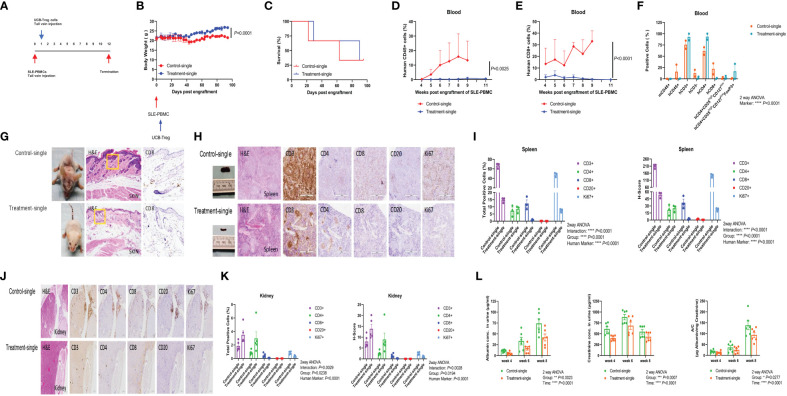
Single injection of CD4^+^CD25^+^CD127^low^ UCB-Tregs halts disease progression in SLE xenografts. **(A)** Schematic summary of single injection of CD4^+^CD25^+^CD127^low^ UCB-Treg cell therapy for SLE in a SLE xenograft model. Female Rag2^-/-^ γc^-/-^ mice were transplanted with 3 × 10^6^ human SLE-PBMCs by intravenous injection on day 0 and divided into two groups (Control-single and Treatment-single, *n* = 3 per group). Single injection of 10 × 10^6^ *ex vivo* expanded CD4^+^CD25^+^CD127^low^ UCB-Tregs was administered through t.v. on day 7 after SLE-PBMCs injection. **(B)** CD4^+^CD25^+^CD127^low^ UCB-Tregs improve body weight in SLE xenografts. Change of body weight was monitored twice per week until termination of experiments. Data are presented as mean ± SEM (*n* = 3). *p* < 0.05 by Student *t-*test was considered statistically significant. **(C)** CD4^+^CD25^+^CD127^low^ UCB-Tregs improves median survival days. Kaplan–Meier analysis was performed to estimate median survival times. **(D)** CD4^+^CD25^+^CD127^low^ UCB-Tregs decrease human CD45^+^ cells. Mouse PBMC was procured from Control-single and Treatment-single recipients and analyzed for CD45^+^ cells as measured by flow cytometry at the indicated time points. *p* < 0.05 by two-tailed unpaired Student *t-*test was considered statistically significant. **(E)** CD4^+^CD25^+^CD127^low^ UCB-Tregs decrease human CD8^+^ cells. Mouse PBMC was procured from Control-single and Treatment-single recipients and analyzed for CD8^+^ cells as measured by flow cytometry at the indicated time points. Data are presented as mean ± SEM. *p*-values were obtained using two-tailed unpaired *t-*test with 95% confidence interval for evaluation of statistical significance compared with the untreated controls. *p* < 0.05 was considered statistically significant. **(F)** Comparison of phenotypes between Control-single and Treatment-single recipients at 8 weeks post engraftment of SLE-PBMCs. *p* < 0.05 was considered statistically significant. *p* < 0.0001 by one-way ANOVA test. **(G)** Photograph of representative mouse Control-single and Treatment-single arm exhibiting skin changes. Representative H&E and CD8 staining of mouse-affected skin tissue sections from Control-single and Treatment single arm. **(H)** Photograph of spleen and representative H&E, CD3, CD4, CD8, CD20, and Ki67 staining of spleen tissue sections from representative mouse Control-single and Treatment-single arm. **(I)** Quantification analysis of positive cells and H-score of spleen tissue sections. Immunochemistry images of human CD3^+^, CD4^+^, CD8^+^, CD20^+^, and Ki67^+^ cells were analyzed at ×40 magnification using HALO 3.3 software. Quantification analysis of human CD3^+^, CD4^+^, CD8^+^, CD20^+^, and Ki67^+^ cells and H-scores for human CD3, CD4, CD8, CD20, and Ki67 positivity were calculated using HALO 3.3 software. Data are presented as mean ± SEM (*n* = 3). *p* < 0.05 was considered statistically significant. ****p < 0.0001 by two-way ANOVA with Tukey multiple comparison tests. **(J)** Single injection of CD4^+^CD25^+^CD127^low^ UCB-Tregs decreased renal inflammation *in vivo*. Representative H&E, CD3, CD4, CD8, CD20, and Ki67 staining of kidney tissue sections. **(K)** Quantification analysis of positive cells and H-score of kidney tissue sections. Immunochemistry images of human CD3^+^, CD4^+^, CD8^+^, CD20^+^, and Ki67^+^ cells were analyzed at ×40 magnification using HALO 3.3 software. Quantification analysis of human CD3^+^, CD4^+^, CD8^+^, CD20^+^, and Ki67^+^ cells and H-scores for human CD3, CD4, CD8, CD20, and Ki67 positivity were calculated using HALO 3.3 software. Data are presented as mean ± SEM (n=5). *p* < 0.05 was considered statistically significant. **(L)** Single injection of CD4^+^CD25^+^CD127^low^ UCB-Tregs decreased albuminuria in SLE xenografts. Expression levels of urinary albumin, creatinine, and albumin/creatinine. Data are presented as mean ± SEM (*n* = 6). *p* < 0.05 was considered statistically significant. *p < 0.05, **p < 0.01, ***p < 0.001, ****p < 0.0001 by two-way ANOVA with Tukey multiple comparison tests.

SLE xenograft was established as described previously (25), where single t.v. injection of CD4^+^CD25^+^CD127^low^ UCB-Tregs was administered at 7 days after SLE-PBMC injection (**Figure 3A**). When compared to SLE-PBMC only recipients (Control-single), a single injection of CD4^+^CD25^+^CD127^low^ UCB-Tregs (Treatment-single) led to an improvement in weight gain (**Figure 3B**) and survival (63 median survival days for the Control-single group and 90 median survival days for the Treatment-single group, **Figure 3C**). At 11 weeks, the Treatment-single group demonstrated a significant reduction in the circulating human CD45^+^ cells (*p* = 0.0025; **Figure 3D**) and human CD8^+^ T cells (*p* < 0.0001; **Figure 3E**). At 8 weeks, Treatment-single demonstrated an increase in circulating human CD4^+^ cells (93.4% vs. 61.7%) and CD4^+^CD25^high^CD127^low^FoxP3^+^ cells (16.7% vs. 0.8%) (**Figure 3F**). Such an increase in the CD4^+^ cell populations was not evident at the 11-week time point (data not shown).

Widespread skin erythema seen in the Control-single group (**Figure 3G**, left upper panel) that correlated with histologic findings of widespread parakeratosis (**Figure 3G**, middle upper panel) resolved upon the addition of CD4^+^CD25^+^CD127^low^ UCB-Tregs (**Figure 3G**, left lower panel), which correlated with preservation of the epidermal and subdermal layer, and resolution of the CD8^+^ cell infiltrate and intact adipose tissue layer (**Figure 3G**, middle and right lower panel) in the Treatment-single group. Bulky spleen with histologic evidence of lymphoid (white pulp) hyperplasia in the Control-single mice (**Figure 3H**, upper panel) was resolved with the addition of CD4^+^CD25^+^CD127^low^ UCB-Tregs (**Figure 3H**, lower panel) that occurred in conjunction with a reduction of CD3^+^, CD8^+^, CD20^+^, and Ki67^+^ cells as detected by the immunohistochemical (IHC) staining of spleen tissue of the Treatment-single group. Quantification of the IHC staining also showed a significant reduction in both human marker and the H-score for the respective stains (*p* < 0.0001) (**Figure 3I**). As shown in **Figure 3J**, extensive lymphoid infiltrate involving parenchyma and hilar stroma in the kidney tissue of the Control-single group (left upper panel) was notably decreased by the single injection of CD4^+^CD25^+^CD127^low^ UCB-Tregs (Treatment-single group) (left lower panel), with preservation of the renal glomeruli. Additionally, a decrease in the total IHC-positive cells and H-score for human CD8, CD20, and Ki67 marker was also observed in the Treatment-single group whereas both total CD3^+^ and CD4^+^ cells were increased in the Treatment-single group. Group for total positive cells (*p* = 0.0238) and H-score (*p* = 0.0194) was significant. Furthermore, human marker for both total positive cells and H-score was significant (*p* < 0.0001) (**Figure 3K**). Concurrently, a significant improvement in albuminuria (Group: *p* = 0.0023; Time: *p* < 0.0001), creatinuria (Group: *p* = 0.0007; Time: *p* < 0.0001), and urine albumin/creatinine (A/C) ratio (Group: *p* = 0.0277; Time: *p* < 0.001) was also demonstrated (**Figure 3L**).

### Multiple injections of CD4^+^CD25^+^CD127^low^ UCB-Tregs can resolve SLE pathology

**Figure 4:**
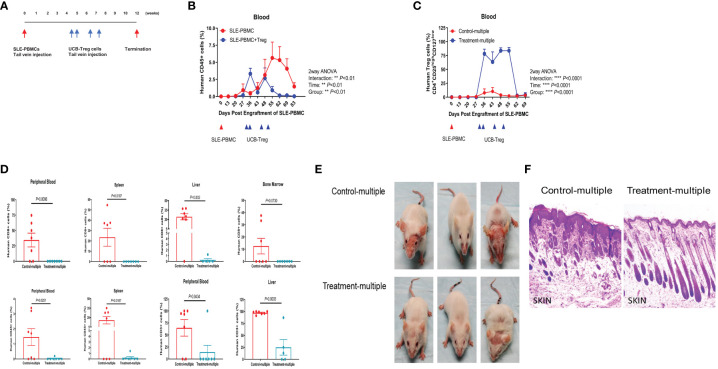
Multiple injections of CD4^+^CD25^+^CD127^low^ UCB-Tregs increase Tregs, decrease CD8^+^ T cells, and improve skin inflammation in the SLE xenogeneic model. **(A)** Schematic summary of multiple CD4^+^CD25^+^CD127^low^ UCB-Treg cell therapy for SLE in a SLE xenograft model. Female Rag2^-/-^ γc^-/-^ mice were transplanted with 3 × 10^6^ human SLE-PBMCs by intravenous injection. After mice displayed human immune cells, they were divided into two groups (control and treatment, *n* = 7 mice/group), and 10 × 10^6^ *ex vivo* expanded CD4^+^CD25^+^CD127^low^ UCB-Tregs were infused into SLE xenografts intravenously on day 30, day 35, day 45, and day 51 for treatment. **(B)** Sustained decrease in PB human CD45^+^ cells in CD4^+^CD25^+^CD127^low^ UCB-Treg recipients. Mouse PBMC was procured from Control-multiple and Treatment-multiple recipients and analyzed for CD45^+^ cells by flow cytometry at the indicated time points. Data are presented as mean ± SEM (*n* = 7). *p* < 0.05 was considered statistically significant. **p < 0.01 by two-way ANOVA with Tukey multiple comparison tests. **(C)** Sustained increase in PB human CD4^+^CD25^high^CD127^low^ Treg cells in CD4^+^CD25^+^CD127^low^ UCB-Treg recipients. Mouse PBMC was procured from Control-multiple and Treatment-multiple recipients and analyzed for CD4^+^CD25^high^CD127^low^ Treg cells by flow cytometry at the indicated time points. Data are presented as mean ± SEM (*n* = 7). *p*-values were obtained using two-way analysis of variance (ANOVA) with Tukey multiple comparison test. *p* < 0.05 was considered statistically significant. **** *p* < 0.0001 by two-way ANOVA with Tukey multiple comparison tests. **(D)** CD4^+^CD25^+^CD127^low^ UCB-Tregs decrease CD8^+^ T, CD45^+^, and CD3^+^ T cells in multiple organs. At the time of euthanasia, PB and organs from Control-multiple and Treatment-multiple recipients were harvested and liquified and analyzed for human CD8^+^ T cells, CD45^+^ cells, and CD3^+^ T cells by flow cytometry at the indicated time points. Data are presented as mean ± SEM (*n* = 5-7). *p* < 0.05 by Student *t-*test was considered statistically significant. **(E)** CD4^+^CD25^+^CD127^low^ UCB-Tregs decrease skin disease burden in SLE xenografts. Photographs of Control-multiple (upper panel) and Treatment-multiple (lower panel) were compared at 12 weeks. **(F)** Representative H&E staining of mouse skin tissue sections of Control-multiple (epidermal ulceration, subdermal lymphocyte infiltrate, disruption of hair follicles, and loss of subdermal adipose tissue) and Treatment-multiple (epidermal layer intact and clear visualization of the subdermal layers including hair follicles and adipose tissue).

Using the same SLE xenograft model, we allowed for 4 weeks to establish human disease in immune-deficient mice followed by multiple t.v. injections of CD4^+^CD25^+^CD127^low^ UCB-Tregs administered on days 30, 35, 45, and 51 (**Figure 4A**). The Treatment-multiple group demonstrated a significant reduction in the circulating human CD45^+^ cells (**Figure 4B**, *p* < 0.01). The percentage of circulating CD4^+^CD25^high^CD127^low^ Treg cells was significantly increased following multiple CD4^+^CD25^+^CD127^low^ UCB-Tregs injections and lasted up to day 62 (**Figure 4C**, *p* < 0.0001). Mice were euthanized at 12 weeks where the phenotype analysis of PB and aseptically harvested single cell organ suspensions showed a significant decrease in human CD8^+^ T cells in PB (*p* = 0.0096), spleen (*p* = 0.0187), and liver (*p* = 0.002); human CD45^+^ cells in PB (*p* = 0.0251) and spleen (*p* = 0.0187); and human CD3^+^ cells in PB (*p* = 0.0434) and liver (*p* = 0.0003) in the treatment group (Treatment-multiple), when compared to the control arm (Control-multiple) (**Figure 4D**). Mice in the control group developed malar, discoid and erythematous skin rash, and/or hair loss (**Figure 4E**, upper panel), similar to that observed in human disease (27). Such aggressive cutaneous manifestations were not observed in the treatment group (Treatment-multiple) (**Figure 4E**, lower panel). Histopathological examination of affected skin biopsy of control mice (Control-multiple) showed lymphocytic infiltrate, subdermal edema, loss of hair follicles, and widespread parakeratosis and hair follicle loss when compared to intact hair follicles and preservation of the subdermal layer in the tissue obtained from the affected site of the Treatment-multiple group mouse (**Figure 4F**).

**Figure 5:**
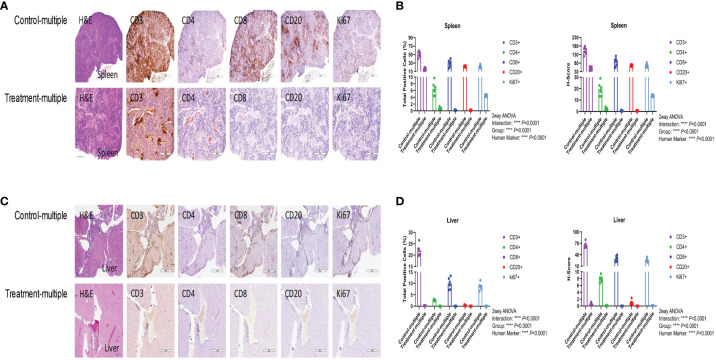
CD4^+^CD25^+^CD127^low^ UCB-Tregs decrease tissue inflammation in SLE xenografts. To assess pathology and cellular infiltrates in the tissue, human CD3^+^ cells, CD4^+^ T cells, CD8^+^ T cells, CD20^+^ B cells, or Ki67^+^ cells in the spleen **(A, B)** and liver **(C, D)** of untreated (Control-multiple) and treated mice (Treatment-multiple) were detected by immunohistochemistry. Spleen **(A)** and liver **(C)** tissues were harvested, fixed with 10% buffered formalin, and embedded in paraffin for processing into 5-μm tissue sections. To assess pathology and cellular infiltrates in the tissues, de-paraffinized and rehydrated tissue sections were stained with Hematoxylin & Eosin. For immunohistochemistry, de-paraffinized and rehydrated tissue sections were subjected to heat-mediated antigen retrieval with sodium citrate buffer (pH 6), permeabilization, and blocking prior to staining with primary antibodies against human CD3, CD4, CD8, CD20, and Ki-67, and appropriate horseradish peroxidase-conjugated secondary antibodies were used to determine the subset of human cells in the mouse tissues. Immunochemistry images of human CD3^+^, CD4^+^, CD8^+^, CD20^+^, and Ki67^+^ cells were analyzed at ×40 magnification using HALO 3.3 software in spleen **(B)** and liver **(D)**. Quantification analysis of human CD3^+^, CD4^+^, CD8^+^, CD20^+^, and Ki67^+^ cells and H-scores for human CD3, CD4, CD8, CD20, and Ki67 positivity were calculated using HALO 3.3 software. Data are presented as mean ± SEM (*n* = 6). *p* < 0.05 was considered statistically significant. *p* < 0.0001 by two-way ANOVA with Tukey multiple comparison tests.

As shown in **Figure 5A**, histopathological examination of the spleen showed diffuse lymphocytic infiltration with loss of red- and white-pulp demarcation in the Control-multiple group, whereas spleen tissue architecture was well preserved in the Treatment-multiple group. The total number of human CD3^+^, CD4^+^, CD8^+^, CD20^+^, and Ki67^+^ cells and the H-score for spleen IHC stains were significantly decreased in the Treatment-multiple group compared with the Control-multiple group (*p* < 0.0001) (**Figure 5B**). A significant reduction in the total number of human CD3^+^, CD4^+^, CD8^+^, CD20^+^, and Ki67^+^ cells and the H-score for all liver IHC stains in the Treatment-multiple group was also observed (*p* < 0.0001) (**Figures 5C, D**).

### CD4^+^CD25^+^CD127^low^ UCB-Tregs improve renal function and decrease disease activity

**Figure 6:**
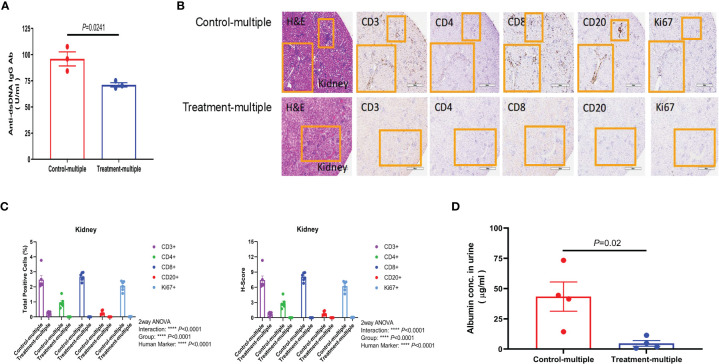
CD4^+^CD25^+^CD127^low^ UCB-Tregs decrease anti-dsDNA IgG Ab and improve renal function in SLE xenografts. **(A)** Multiple injections of CD4^+^CD25^+^CD127^low^ UCB-Tregs decrease anti-human dsDNA Ab. Plasma samples were collected from the EDTA-treated PB of untreated (Control-multiple) and CD4^+^CD25^+^CD127^low^ UCB-Tregs-injected mice (Treatment-multiple). Levels of anti-human dsDNA IgG Ab were measured using the Abnova assay kit. Data are presented as mean ± SEM (*n* = 3). *P*-values were obtained using two-tailed unpaired *t-*test with 95% confidence interval for evaluation of statistical significance compared with the untreated controls. *P* < 0.05 was considered statistically significant. **(B)** Representative H&E, CD3, CD4, CD8, CD20, and Ki67 staining of kidney tissue sections of Control-multiple (upper panel) and Treatment-multiple (lower panel). **(C)** Quantification analysis of human CD3^+^, CD4^+^, CD8^+^, CD20^+^, and Ki67^+^ cells and H-scores for human CD3, CD4, CD8, CD20, and Ki67 positivity. Total positive cells and H-score were calculated using HALO 3.3 software. Data are presented as mean ± SEM (*n* = 6). *p* < 0.05 was considered statistically significant. ****p < 0.0001 by two-way ANOVA with Tukey multiple comparison tests. **(D)** CD4^+^CD25^+^CD127^low^ UCB-Tregs decrease albuminuria in SLE xenografts. At the time of euthanasia, at 12 weeks, urine samples were collected from Control-multiple and Treatment-multiple and levels of urinary albumin were assessed using the Exocell Albumin M assay kit. Data are presented as mean ± SEM (*n* = 4). *p*-values were obtained using two-tailed unpaired *t-*test with 95% confidence interval for evaluation of statistical significance compared with the untreated controls. *p* < 0.05 was considered statistically significant.

Since anti-double-stranded DNA (dsDNA) antibodies are known to contribute to pathogenesis of LN (28), we measured their levels in the Control-multiple and Treatment-multiple arm. As shown in **Figure 6A**, multiple injections of CD4^+^CD25^+^CD127^low^ UCB-Tregs significantly reduced circulating levels of human anti-dsDNA IgG antibody at 12 weeks (*p* = 0.0241). Histopathological evaluation of kidney tissue at the time of euthanasia revealed intense lymphocytic infiltrate in the Control-multiple arm compared to preservation of tissue architecture in the Treatment-multiple arm (**Figure 6B**, left panel). IHC staining of renal tissue showed a significant decrease in the total number of CD3^+^, CD4^+^, CD8^+^, CD20^+^, and Ki67^+^ cells and the H-score for all kidney IHC stains in the Treatment-multiple arm when compared to the Control-multiple arm (*p* < 0.0001) (**Figures 6B, C**). Since SLE-induced renal inflammation leads to organ dysfunction as captured by albuminuria (29), we measured secretion of albumin in mouse urine. As shown in **Figure 6D**, a significant reduction in albuminuria was observed in the Treatment-multiple arm when compared to the Control-multiple arm (*p* = 0.02).

### CD4^+^CD25^+^CD127^low^ UCB-Tregs resolve SLE inflammation *in vivo*

**Figure 7:**
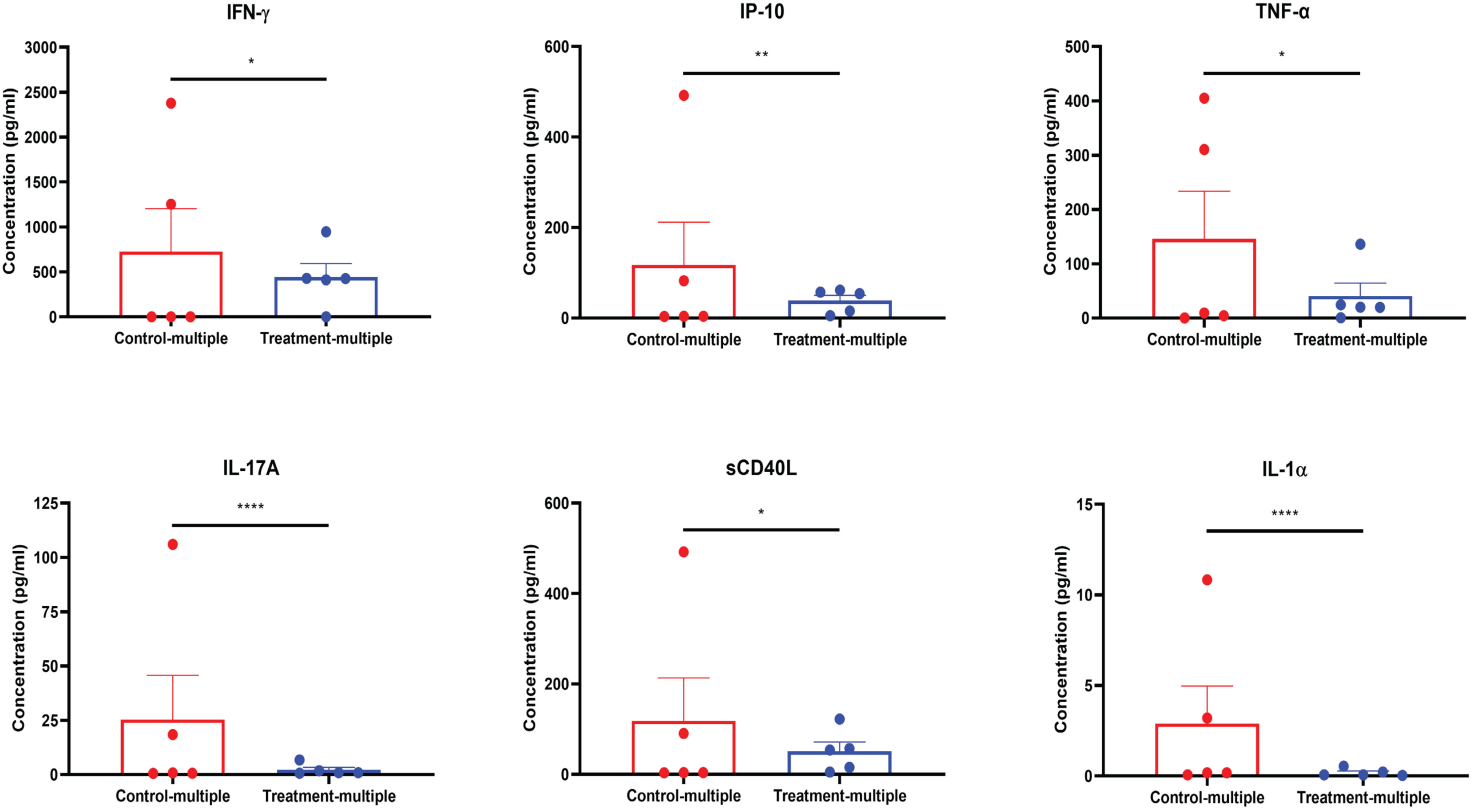
CD4^+^CD25^+^CD127^low^ UCB-Tregs decrease systemic inflammation in SLE xenografts. Plasma samples were collected from the EDTA-treated PB of untreated (Control-multiple) and CD4^+^CD25^+^CD127^low^ UCB-Tregs-infused mice (Treatment-multiple). Levels of human IFN-γ, IP-10, TNF-α, IL-17A, sCD40L, and IL-1α in the plasma of SLE xenografts were measured using the Human cytokine 42-plex Discovery assay kit. Data are presented as mean ± SEM (*n* = 5). *p* < 0.05 by *F*-test was considered statistically significant. * *p* < 0.05, ** *p* < 0.01, **** *p* < 0.0001 by two-way ANOVA with Tukey multiple comparison tests.

As shown in **Figure 7**, at the time of euthanasia, mouse plasma showed a reduction in the levels of circulating inflammatory cytokines including IFN-γ, IP-10, TNF-α, IL-17A, sCD40L, and IL-1α, which overlapped with those impacted in *in vitro* studies (**Figures 2B–I**).

## Discussion

Here, we provide proof of concept of using adoptive therapy with CD4^+^CD25^+^CD127^low^ UCB-Tregs for the treatment of LN by decreasing systemic and renal inflammation, and overall disease burden as shown by a decrease in anti-dsDNA Ab and improvement in albuminuria. The superiority of CD4^+^CD25^+^CD127^low^ UCB-Tregs is highlighted by their ability to suppress both pathogenic SLE cells and healthy donor Tcon cells, to a similar degree, and thus provides proof that CD4^+^CD25^+^CD127^low^ UCB-Tregs are not impacted by the potential Teff resistance, well described as a possible point of failure in using CD4^+^CD25^+^CD127^low^ Treg adoptive therapy in SLE (14). At a fundamental level, the co-culture of CD4^+^CD25^+^CD127^low^ UCB-Tregs with SLE-PBMC shifts the dominance of CD8^+^ Teffs and CD19^+^ lupus B cells towards the CD4^+^CD25^+^CD127^low^ Treg phenotype and CD4^+^CD8^+^ dual expressing T cells, which have been shown to have a suppressive effect on the production of autoantibodies including anti-dsDNA Ab in SLE (30). CD4^+^CD25^+^CD127^low^ UCB-Tregs decrease the percentage of pathogenic monocytes, which have been shown to be associated with deterioration of kidney function in lupus patients (31). In fact, our CD4^+^CD25^+^CD127^low^ UCB-Tregs were able to continue their IL-10 secretion, a well-described suppressive cytokine, to inhibit the proliferation of pathogenic SLE-PBMCs *in vitro*. IL-2-dependent IL-10 expression in human CD4^+^CD25^+^CD127^low^ Tregs has been shown to be mediated by Stat5 recruitment to a Stat-binding motif in the fourth intron of IL-10 (I-SRE) (32), which can, in turn, induce suppression of Teffs (33). Recently, efforts have been made to use external IL-2 injection to increase CD4^+^CD25^+^CD127^low^ Treg cells *in vivo* in lupus patients (34–36). However, our data do not support such an approach since the stimulation of SLE-PBMCs with IL-2 and CD3/28 beads did increase in CD4^+^ CD25^+^CD127^low^ Treg cell population, but it did not translate into suppressor function as there was no increase in the secretion of IL-10. Therefore, such an acquired phenotype may not correlate with functional CD4^+^CD25^+^CD127^low^ Treg cells and caution must be exercised when evaluating systemic IL-2 drug treatment as an attempt to stimulate and/or increase CD4^+^CD25^+^CD127^low^ Tregs in patients suffering from SLE and other autoimmune diseases, since the correlation of *in vivo* expansion of CD4^+^CD25^+^CD127^low^ Treg cells in response to low-dose IL-2 in SLE patients with a clinical response in open-label studies did not translate into clinical efficacy in randomized controlled trials (37). In fact, stimulation of human CD4^+^CD25^+^CD127^low^ Treg cells under inflammatory conditions in the presence of IL-2 carries the risk of converting them into TH17 cells (38).

Co-culture with CD4^+^CD25^+^CD127^low^ UCB-Tregs also decreased the percentage of CD56^+^ NK cells in the SLE-PBMC population, implicated in excessive IFN-γ production in patients with active SLE (39), where elevated levels of IFN-γ have been shown to be associated with nephrotic syndrome (40). Independently, co-culture with our CD4^+^CD25^+^CD127^low^ UCB-Tregs significantly decreased IFN-γ secretion by the pathogenic lupus cells *in vitro* and multiple injections of CD4^+^CD25^+^CD127^low^ UCB-Tregs in SLE xenografts decreased circulating IFN-γ levels with a corresponding improvement in kidney function. Recently, the role of IFN-γ and IFN-γ-inducible GBP1 gene has been shown to mediate the development of SLE in a gene expression study (41). IFN-γ is a major proinflammatory cytokine that regulates the functions of several important immune system cells, including B cells and T cells (42), directly inhibits CD4^+^CD25^+^CD127^low^ Treg cell function (43), and contributes significantly to the development of SLE (44). Our CD4^+^CD25^+^CD127^low^ UCB-Treg cells were able to overcome the inhibitory effect of IFN-γ, which may additionally contribute to its multi-dimensional mechanism of action as a therapeutic agent for SLE patients.

CD4^+^CD25^+^CD127^low^ UCB-Tregs decreased CD19^+^ B-cell population in the SLE-PBMCs. B cells have been identified to drive lupus pathogenesis and are the target of currently approved biologics treatment for SLE (45, 46). Recently, CD19-targeted CAR T cells treatment was shown to induce clinical remission with a decrease in proteinuria in a patient with refractory SLE, where the expansion of CAR T cells preceded the complete and sustained depletion of circulating B cells, resulting in a decrease in anti-dsDNA autoantibodies level (47). A similar coupled decrease of anti-dsDNA IgG Ab and albuminuria was also observed *in vivo* in response to treatment with multiple injections of our CD4^+^CD25^+^CD127^low^ UCB-Tregs.

The active role of monocytes in accelerating inflammation and injury in kidney glomerular lesions has been identified in SLE (48, 49). Monocytes in SLE have been shown to engage the CD40/CD40L signaling pathways to contribute to lupus pathogenesis (50). Our CD4^+^CD25^+^CD127^low^ UCB-Tregs decreased monocytes in the SLE-PBMC population as well as reduced soluble CD40L *in vitro* and *in vivo*. Additionally, CD4^+^CD25^+^CD127^low^ UCB-Tregs also decreased several inflammatory cytokines implicated in lupus pathogenesis both *in vitro* and *in vivo*, including IP-10 (51), TNF-α (52), IL-6 (53), IL-17A (54), and IL-1 (55). The crosstalk between the circulating and tissue-resident cytotoxic CD8^+^ T cells and widespread tissue destruction including skin, spleen, and kidney, as well as systemic and tissue inflammation, clearly evident in our xenogeneic lupus model, is supported by the published data where local cytokine, chemokine, and adhesion molecule production has been shown to encourage the further influx of inflammatory cells and the production of proinflammatory cytokines, ultimately resulting in tissue inflammation, tissue injury, and, eventually, fibrosis (56).

The remarkable ability of our CD4^+^CD25^+^CD127^low^ UCB-Treg cells to interrupt the vicious inflammation–injury loop provides proof of concept of their activity in SLE. Previous reports have shown the role of anti-dsDNA isotypes and anti-C1q antibody in the diagnosis of SLE and their association with disease activity and LN (57, 58). Our results suggest that the correlation of CD4^+^CD25^+^CD127^low^ UCB-Tregs administration with decreases in known disease measures such as anti-dsDNA IgG Ab as well as the quantification of the end organ damage including albuminuria and its multimodal mechanism of action makes them ideal for the treatment of LN.

## Data availability statement

The raw data supporting the conclusions of this article will be made available by the authors, without undue reservation.

## Ethics statement

The studies involving humans were approved by The University of Texas M.D. Anderson Cancer Center Institutional Review Board. The studies were conducted in accordance with the local legislation and institutional requirements. The animal study was approved by The University of Texas M.D. Anderson Cancer Center Institutional Animal Care & Use Committee. The study was conducted in accordance with the local legislation and institutional requirements.

## Author contributions

Conception and design of the study: M-AL and SP. Acquisition, analysis, and interpretation of data: M-AL, XT, JDK, MGR, MH, MN, KZ, HM, CRF, and SP. Drafting and revising the manuscript: M-AL and SP. All authors contributed to the article and approved the submitted version.
